# Supplementary material for: Characterization of Two Novel Insect-Specific Viruses Discovered in the Green Leafhopper, Cicadella viridis
Source: Insects. 2022 Apr 12;13(4):378. doi: 10.3390/insects13040378 (PMC9032321; doi:10.3390/insects13040378)
Supplement: Supplementary file 1 [file insects-13-00378-s001.zip › Figure S1&2.pdf]

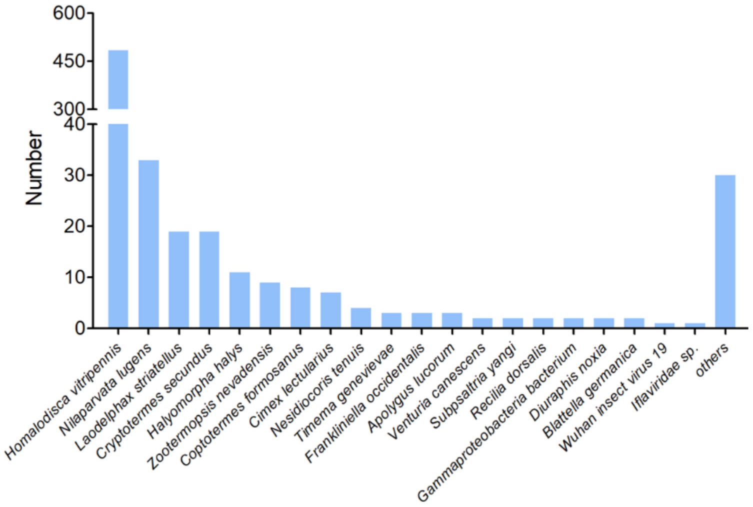

Figure S1. Species distribution of the BLASTX results of 674 contigs. Contigs that show sequence similarity to viral proteins (cutoff E-value of 1×10^{-20}) were BLAST against the entire NCBI databases, and the best matched species are summarized.

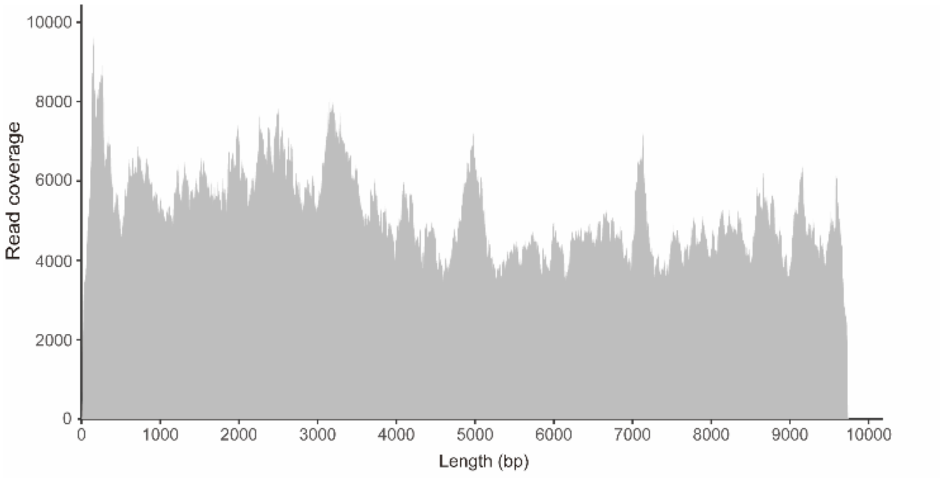

Figure S2. Rearrangement of RNA-seq reads to Cicadella viridis iflavirus 1 (CvIfV1) genome. The RNA-seq dataset of *C. viridis* (accession number: SRR11729954) was retrieved from NCBI SRA repository, and mapped to CvIfV1 genome.
