# Supplementary material for: Characterization of Two Novel Insect-Specific Viruses Discovered in the Green Leafhopper, Cicadella viridis
Source: Insects. 2022 Apr 12;13(4):378. doi: 10.3390/insects13040378 (PMC9032321; doi:10.3390/insects13040378)
Supplement: Supplementary file 1 [file insects-13-00378-s001.zip › Table S1 Primers used in this study.pdf]

Supplementary Table S1. Primers used in this study.

Primer	Sequence (5' to 3')	Purpose
Long primer	CTAATACGACTCACTATAGGGCAAGCAGTGGTAT CAACGCAGAGT	Amplification of 5′/3′ RACE fragment
Short primer	CTAATACGACTCACTATAGGGC	
5′-RACE GSP	TGTAGGTCTGTATCTTCGTCTC	Amplification of 5′ RACE fragment of CvIfV1
3′-RACE GSP	GTCTGTGTCTATGGAGATGACCTGAT	Amplification of 3′ RACE fragment of CvIfV1
5′-RACE GSP	TCTGTACCATTCACTGTTGTCTG	Amplification of 5′ RACE fragment of CvNiLV1
3′-RACE GSP	GTATAGCGCATTGCAGCCGGACG	Amplification of 3′ RACE fragment of CvNiLV1
CvIfV1-F	TGTACGAATTGACGCAGGAGGAGGAT	Amplification of CvIfV1 viral genome
CvIfV1-R	GTCTAGAAGGCGATACGCCAGCGGC	
CvNiLV1-F	CAACTGCAGATGAGGTAGAGGATCT	
CvNiLV1-R	TGTTCAGGATTTTCAACAGCATTT	
CvNiLV1-F	AGTTGTCGCGGGTTTAGAGAAGC	Amplification of CvIfV1 viral genome
CvNiLV1-R	CCCTTCCTTCAAAGTCAGACAATC	
CvNiLV1-F	TGACAGTACTAGTGGCAAAAGTA	

CvNiLV1-R	GGATCATATCTACGCATCGTGCA	Amplification of CvIfV1 viral genome

Abbreviations: GSP, Gene Specific Primer; Cicadella viridis iflavirus 1, CvIfV1; Cicadella viridis nido-like virus 1, CvNiLV1.
